# MRI changes observed in a case of atypical scrapie in a 7-year-old Herdwick ewe

**DOI:** 10.1177/10406387241267849

**Published:** 2024-09-06

**Authors:** Sander Prins, Kim Hamer, Ana Cloquell, John Spiropoulos, Neil Sargison, Piet Vellema

**Affiliations:** School of Biodiversity, One Health and Veterinary Medicine, University of Glasgow, Glasgow, UK; School of Biodiversity, One Health and Veterinary Medicine, University of Glasgow, Glasgow, UK; School of Biodiversity, One Health and Veterinary Medicine, University of Glasgow, Glasgow, UK; Animal and Plant Health Agency–Weybridge, New Haw, UK; Royal (Dick) School of Veterinary Studies, University of Edinburgh, Easter Bush, Midlothian, UK; Department of Small Ruminant Health, Royal GD, Deventer, the Netherlands

**Keywords:** atypical scrapie, immunohistochemistry, magnetic resonance imaging, PrP^Sc^, sheep, transmissible spongiform encephalopathy

## Abstract

Atypical scrapie is a transmissible spongiform encephalopathy that is rarely diagnosed in living animals. In March 2022, a 7-y-old Herdwick ewe was referred to the Scottish Centre for Production Animal Health and Food Safety because of circling behavior and ill thrift. The ewe had a low body condition score, was obtunded, with a wide-based stance of the pelvic limbs, and was circling to the left. Hematologic, biochemical, and CSF analyses were unremarkable, but postmortem magnetic resonance imaging (MRI) findings were consistent with diffuse, bilateral, and symmetrical atrophy of the forebrain and ventriculomegaly. The clinical signs, the involvement of an individual older ewe, and the MRI results led to the clinical diagnosis of scrapie. Immunohistochemistry on the fixed brain, performed by the U.K. Animal and Plant Health Agency, revealed deposits of PrP^Sc^, which is a specific disease marker of transmissible spongiform encephalopathies, mainly in the cerebellum and at lower concentrations in the cerebrum and obex, consistent with the diagnosis of atypical scrapie. MRI findings in a sheep with atypical scrapie have not been described previously, to our knowledge. Scrapie should be included in the list of clinical differential diagnoses when veterinarians are presented with sheep with progressive neurologic signs of several weeks’ duration.

Transmissible spongiform encephalopathies (TSEs) are fatal neurodegenerative diseases that can affect humans and animals. TSEs include scrapie in small ruminants, chronic wasting disease (CWD) in deer, transmissible mink encephalopathy (TME) in mink, bovine spongiform encephalopathy (BSE) in cattle, and Creutzfeldt-Jakob disease (CJD) in humans.^12^ TSEs are caused by the accumulation of PrP^Sc^ in the brain, a pathogenic protein that is a misfolded isoform of the original host prion protein PrP^C^.^30^ In 1998, a different form of scrapie, designated Nor98/atypical scrapie, was identified in 5 Norwegian flocks with the affected animals differing in age and genotype, and with differing molecular profile and location of the PrP^Sc^ compared to classical scrapie.^4,25^ The BSE epidemic in the United Kingdom in the 1990s led to increased interest in TSEs, and active surveillance of TSEs in cattle, sheep, and goats was introduced throughout the European Union from 2002 onwards, under Regulation (EC) no. 999/2001 of the European Parliament in 2001.^10^ TSE surveillance in slaughtered animals and fallen stock resulted in increased detection of atypical scrapie cases.^3,6,8^ Compared to classical scrapie, atypical scrapie is detected less frequently in the EU and UK; in 2022, 557 cases of classical scrapie were detected versus 77 cases of atypical scrapie.^9^

Atypical scrapie is mostly detected through active surveillance in fallen stock or culled animals rather than through passive surveillance; this may suggest that clinical signs are not present at the time animals are sent for slaughter.^17,21^ Alternatively, a veterinarian may not be consulted because so few animals are affected and those affected may be close to the end of their productive life. Affected animals can show signs of ill thrift, circling, hyperesthesia, pelvic limb ataxia, head tremors, and a wide-based stance of the pelvic limbs.^4,14,16^ We describe here the clinical signs and postmortem diagnostic confirmation of atypical scrapie in a 7-y-old ewe, referred for veterinary investigation of neurologic signs.

In March 2022, a 7-y-old Herdwick ewe was referred to the Scottish Centre for Production Animal Health and Food Safety (Glasgow) because it had been showing signs of ill thrift and circling gait for ~6 wk. No treatment had been given prior to hospitalization. The ewe had been introduced to the flock 4 y previously, and no similar signs had been reported in livestock on either the farm of origin or on the farm to which she was introduced.

At presentation, the ewe had a low body condition score (BCS) of 1.5 (on a scale of 0–5), was obtunded, had a wide-based stance of the pelvic limbs, and occasionally circled to the left. Heart rate was 72 bpm and body temperature 39.2°C; both were considered within normal limits.^18^ During hospitalization, the ewe did not eat and spent most of the time standing. Neurologic examination revealed moderate proprioceptive ataxia in all 4 limbs. Occasional circling to the left was observed that increased in intensity when the ewe was approached or touched by humans. Segmental spinal cord reflexes were normal, as was the cranial nerve examination. Neuroanatomic localization was consistent with multifocal intracranial lesions mainly affecting the forebrain. Differential diagnoses included listeriosis, space-occupying lesions, maedi-visna, and scrapie.

The results of biochemical and hematologic testing of blood samples were unremarkable. At this point, the animal was euthanized, and a CSF sample was taken to investigate the presence of a bacterial or viral infection. No significant increase in proteins or nucleated cells was demonstrated. As part of the diagnostic analysis, postmortem magnetic resonance imaging (MRI) of the cranium was performed to evaluate potential intracranial structural causes such as neoplasia, traumatic injuries, cystic lesions, or degenerative diseases.^1,27^

MRI was performed with a 1.5-T magnet (Magentom Essenza 1.5 MRI; Siemens). Only T2-weighted pulse sequences were obtained in the sagittal, transverse, and dorsal planes. Although no structural lesions or mass were observed, MRI images were observed that are associated with brain atrophy (Fig. 1). These include: 1) marked prominence of the cerebral sulci in the frontal, temporal, parietal, and occipital lobes, which was observed by the increased amount of CSF occupying the sulci of the cerebral surface; 2) symmetrical enlargement of the lateral ventricles, including the most rostral part at the level of the olfactory lobes; 3) reduction of the thickness of the forebrain, most obviously noted on the cerebral hemispheres, thalamus, and hippocampus; 4) an abnormally small and dorsally displaced corpus callosum; and 5) suspected reduction of the size of the interthalamic adhesion. No obvious changes were observed in the cerebellum and brainstem. No intra-axial lesions were observed. As the suspected brain atrophy was mild, MRI results were compared with a healthy control case that was also performed postmortem (Fig. 2). Morphometric measurements were performed, demonstrating an Evans index of 0.31 cm, which is increased compared to the reference range in healthy sheep of 0.22 ± 0.04 cm,^29^ confirming the enlargement of the lateral ventricles (Fig. 3A). The lateral ventricle:cerebrum area ratio (V:C ratio) was 14% (Fig. 3B), which is higher than the <10.5% found in normal sheep.^20^

**Figure 1.**
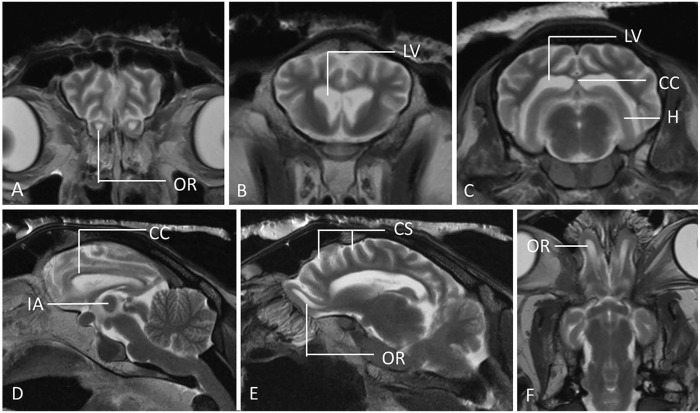
Postmortem MRI sections in T2W transverse (**A–C**), sagittal (**D**), parasagittal (**E**), and dorsal (**F**) planes of a sheep with atypical scrapie. Significant generalized, bilateral, and symmetrical brain atrophy is evident. Findings suggesting brain atrophy: the cerebral sulcal prominence in the frontal, temporal, parietal, and occipital lobes of both hemispheres; the symmetrical enlargement of the lateral ventricles including the olfactory recesses with secondary thinning of the cerebral cortex and hippocampus; the reduced size and dorsal displacement of the corpus callosum; and a suspected reduction of the size of the interthalamic adhesion (5.5 mm in sagittal image). CC = corpus callosum; CS = cerebral sulci; H = hippocampus; IA = interthalamic adhesion; LV = lateral ventricles; OR = olfactory recesses.

**Figure 2.**
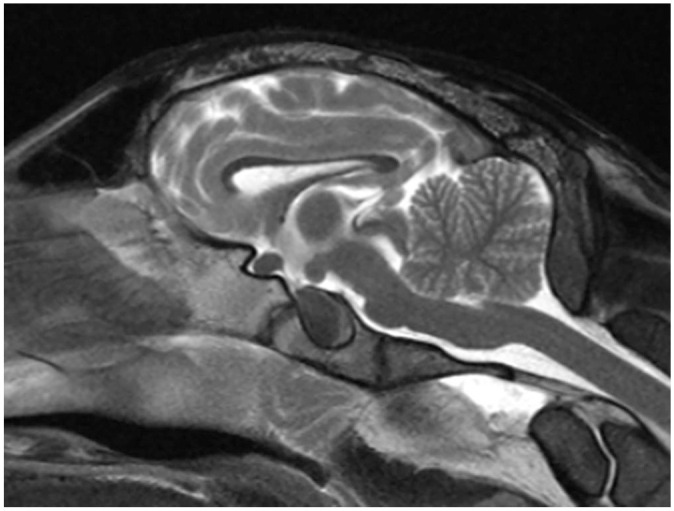
Sagittal postmortem MR image of a normal control sheep. Note the normal size and position of the corpus callosum and the normal size of the interthalamic adhesion.

**Figure 3.**
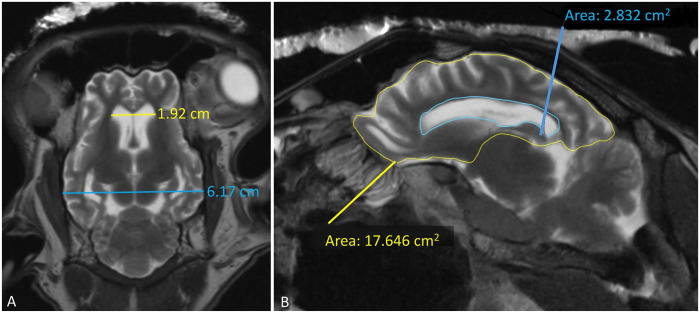
Measurements of the sheep brain were performed on postmortem MRI. **A.** Measurement of the Evans index in a T2W dorsal sequence. The Evans index is calculated as the maximum distance between rostral horns (top line) divided by the maximum internal skull diameter (bottom line) = 0.31 cm in our case (reference in sheep is 0.22 ± 0.04 cm^29^); increased values suggest ventriculomegaly. **B.** Measurement of the ventricle:cerebrum (V:C) ratio was performed as described previously^20^ by outlining the perimeter of the lateral ventricle and the cerebrum on a parasagittal view. The V:C ratio is calculated by the following formula: lateral V:C ratio = lateral ventricle area/(cerebrum area – lateral ventricle area) × 100%, which resulted in 14%.

These clinical and MRI findings suggested a degenerative disorder such as scrapie, therefore formalin-fixed brain tissue was sent to the Animal and Plant Health Agency (APHA) UK (Weybridge) for histopathology (with H&E staining), and immunohistochemistry (IHC) on the frontal, parietal, and occipital cerebrum (including cortical and underlying structures), cerebellum, midbrain, and medulla at the level of the obex. IHC used R145 (APHA Scientific), a rat monoclonal antibody, diluted 1:150. Antigen-antibody complexes were visualized with 3,3′-diaminobenzidine and hematoxylin counterstain.^2^ Extensive PrP^Sc^ deposits were detected in the cerebellum, and to a lesser extent in the cerebrum, midbrain, and obex. Mild vacuolation was also present in the cerebellum (Fig. 4).

**Figure 4.**
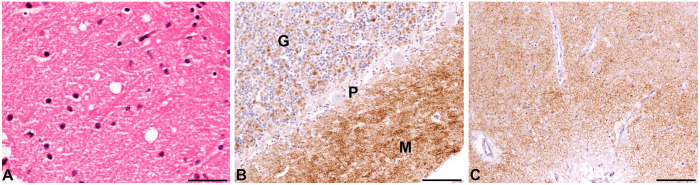
Light microscopy studies of atypical scrapie in a sheep. **A.** Mild TSE-specific vacuolation in the molecular layer of the cerebellar cortex. H&E. Bar = 50 µm. **B.** Immunohistochemistry of the cerebellar cortex. There is prominent granular immunolabeling in the molecular (M) and granular cell (G) layers of the cerebellum. P = Purkinje cell layer. Monoclonal antibody R145. Bar = 100 µm. **C.** Immunohistochemistry of the cerebral cortex. Monoclonal antibody R145. Bar = 100 µm.

Scrapie genotyping on the blood sample that was taken for hematology revealed that the ewe had an ARR/AHQ PrP genotype. A clinical diagnosis of atypical scrapie was given based on the case presentation of a single 7-y-old sheep with a circling gait and ill thrift, but no pruritus, in conjunction with the absence of inflammatory or structural lesions on MRI. The diagnosis of atypical scrapie was confirmed by detection of PrP^Sc^, and the distribution PrP^Sc^ type in the brain. This method produces high-resolution spatial data that can unequivocally discriminate atypical from classical scrapie.^11,15,22^

Based on TSE regulations, the referring farm was placed under restriction for ≥2 y. The restrictions required that all sheep >18-mo-old leaving the farm direct to slaughter or as fallen stock must be tested for TSE. To our knowledge, this is the first description of MRI changes in a case of atypical scrapie. In a search of the Web of Science, Google, PubMed, and CAB Direct, with search terms “MRI”, “atypical scrapie”, and “sheep”, no results were retrieved, which suggests that MRI changes in atypical scrapie have not been reported previously. Similar findings were reported in sheep that were naturally infected with classical scrapie and used as an animal model of CJD.^20^ Of 111 sheep infected with PrP^Sc^, 37 were IHC-positive (28 in brain, 9 in lymphoid tissue only), and all IHC-positive animals had an enlarged V:C ratio compared to the normal V:C in IHC-negative animals, indicating overall brain atrophy in positive animals. Sheep with clinical signs of scrapie had V:C ratios 15% higher than subclinical animals, which may be correlated with disease progression. However, every V:C score >10.4% corresponded to a PrP^Sc^ IHC-positive animal, and every score <9.5% corresponded to a negative animal, which was consistent with our case, which had a V:C ratio of 14%. Another morphometric value, the Evans index, objectively evidenced that the lateral ventricles were enlarged in our case, as it was higher than reported in healthy sheep.^29^ Similar MRI findings have been described in humans with CJD.^20^

The diagnosis of atypical scrapie was based on the clinical signs,^7,16^ the history of involvement of only one individual, an older sheep,^13,19^ and the histopathology, IHC, and MRI findings. By contrast, a clinical diagnosis of classical scrapie would have been more likely if 1) the affected animal had been younger, 2) pruritus was a concomitant clinical sign, and 3) other cases had been seen in the flock. The clinical signs seen in our case are nonspecific for scrapie and can be seen in other diseases: ataxia, weight loss, and wide-based stance of the pelvic limbs, can be seen in maedi-visna,^5^ but the absence of a significant increase in protein in the CSF sample made involvement of the maedi-visna virus less likely.^7,16,28^ The absence of an increase in neutrophils in the CSF made involvement of *Listeria* unlikely.^28^

Brain tissue had been fixed in formalin; hence, our clinical diagnosis of atypical scrapie was confirmed with histology and IHC. The ARR/AHQ genotype found in our case, along with most other heterozygous ARR genotypes, was found in 10–32% of atypical cases in the EU and UK in 2013–2022.^9^

In atypical scrapie, the AHQ and ARR haplotypes are more common and appear to have an increased risk of developing disease.^23,24^ As seen in our case, atypical scrapie is usually found in individual older animals; clinical signs can be vague and difficult to distinguish from other diseases. Nevertheless, reporting through passive surveillance networks is important whenever sheep are presented with dullness, a wide-based stance of the pelvic limbs, or circling gait that persists for several weeks; case investigation enables further research and consideration of potential risk factors.^26^
